# Circular RNA Expression and Regulation Profiling in Testicular Tissues of Immature and Mature Wandong Cattle (*Bos taurus*)

**DOI:** 10.3389/fgene.2021.685541

**Published:** 2021-11-22

**Authors:** Ibrar Muhammad Khan, Hongyu Liu, Jingyi Zhuang, Nazir Muhammad Khan, Dandan Zhang, Jingmeng Chen, Tengteng Xu, Lourdes Felicidad Córdova Avalos, Xinqi Zhou, Yunhai Zhang

**Affiliations:** ^1^ Anhui Provincial Laboratory of Local Livestock and Poultry Genetical Resource Conservation and Breeding, College of Animal Science and Technology, Anhui Agricultural University, Hefei, China; ^2^ Department of Zoology, University of Science and Technology, Bannu, Pakistan

**Keywords:** wandong cattle, testicular growth, spermatogenesis, circRNAs, total RNA sequencing

## Abstract

Wandong cattle are an autochthonous Chinese breed used extensively for beef production. The breed tolerates extreme weather conditions and raw feed and is resistant to tick-borne diseases. However, the genetic basis of testis development and sperm production as well as breeding management is not well established in local cattle. Therefore, improving the reproductive efficiency of bulls *via* genetic selection is crucial as a single bull can breed thousands of cows through artificial insemination (AI). Testis development and spermatogenesis are regulated by hundreds of genes and transcriptomes. However, circular RNAs (circRNAs) are the key players in many biological developmental processes that have not been methodically described and compared between immature and mature stages in *Bovine* testes. In this study, we performed total RNA-seq and comprehensively analyzed the circRNA expression profiling of the testis samples of six bulls at 3 years and 3 months of developmental age. In total, 17,013 circRNAs were identified, of which 681 circRNAs (*p*-adjust < 0.05) were differentially expressed (DE). Among these DE circRNAs, 579 were upregulated and 103 were downregulated in calf and bull testes. The Gene Ontology (GO) and Kyoto Encyclopedia of Genes and Genomes (KEGG) pathway enrichment analyses revealed that the identified target genes were classified into three broad functional categories, including biological process, cellular component, and molecular function, and were enriched in the lysine degradation, cell cycle, and cell adhesion molecule pathways. The binding interactions between DE circRNAs and microRNAs (miRNAs) were subsequently constructed using bioinformatics approaches. The source genes *ATM*, *CCNA1*, *GSK3B*, *KMT2C*, *KMT2E*, *NSD2*, *SUCLG2, QKI*, *HOMER1*, and *SNAP91* were found to be actively associated with bull sexual maturity and spermatogenesis. In addition, a real-time quantitative polymerase chain reaction (RT-qPCR) analysis showed a strong correlation with the sequencing data. Moreover, the developed model of *Bovine* testes in the current study provides a suitable framework for understanding the mechanism of circRNAs in the development of testes and spermatogenesis.

## Introduction

The local Wandong cattle are viewed as one of the primary breeds in Anhui, China, and moreover, they are considered as one of the most economically important breeds in the beef industry. However, their development and utilization are still in the initial stages (Zhou et al., 2001). The reproductive efficiency of breeding bulls is an important consideration in livestock production systems, and the genetic mainstream is obtained through the selective application of germ cells from the desirable sire (Waqas et al., 2019). Hence, molecular strategies are required to improve the reproduction traits and to explore the genetic potential for economically important traits in local cattle breeds.

Mammalian spermatogenesis can be divided into three distinct stages: i) self-renewal and mitosis in spermatogonia to form spermatocytes, ii) meiosis in spermatocytes to generate elongated haploid spermatids, and iii) postmeiotic modifications in haploid spermatids to form spermatozoa (Hecht, 1998; Griswold, 2016; Ibtisham et al., 2017). The crucial stages of spermatogenesis are regulated at the transcriptional, post-transcriptional, and epigenetic levels by a well-coordinated genomics network (Eddy and O'Brien, 1997; Yu et al., 2003). Male gonads consist of complicated transcriptomic elements, with more than 15,000 differentially expressed genes (DEGs) involved in spermatogonial maturation (Ramsköld et al., 2009; Soumillon et al., 2013). Recent studies have demonstrated that noncoding RNAs (nc-RNAs) play an important role at the post-transcriptional level during spermatogenesis (Mukherjee et al., 2014; Robles et al., 2017; Gao et al., 2019).

Circular RNAs (circRNAs) are nc-RNAs with a circular closed loop and play a vital regulatory role in many biological processes. The circRNAs are resistant to RNase degradation because of the lack of 5′- and 3′-ends (Sanger et al., 1976; Lasda and Parker, 2014). In the modern era of bioinformatics and sequencing technology, a significantly high number of circRNAs have been revealed in the mouse brain and testis. It has been shown that the testis consists of the second highest number of tissue-specific circRNA-level genes (You et al., 2015). A total of 15,996 circRNAs have been identified in humans, of which 10,792 (67%) have been observed in the testis (Guo et al., 2014; Dong et al., 2016). Remarkably, a primary report has shown that 1,017 circRNA-linked genes are mostly related to spermatogenesis, sperm motility, and fertilization (Dong et al., 2016). However, little is known about the global profile and characteristics of circRNAs in mammalian testis development and spermatogenesis.

In the present study, we provided a comprehensive catalog of circRNAs in the testes of bulls at two different developmental stages and detailed new insights into the functional activities of circRNAs in testicular development and spermatogenesis. The Gene Ontology (GO) and Kyoto Encyclopedia of Genes and Genomics (KEGG) analyses were performed to ascertain the molecular mechanisms of reproduction that were modulated during growth and development in bulls.

## Materials and Methods

### Animals and Sample Collection

Three physically healthy individuals (*n* = 3) of two different age groups, andrologically mature bulls (3 ± 0.014 years) and immature calves (3 ± 0.24 months), were selected for the current study (Tomlinson et al., 2017). The native cattle are also known as Wandong in the Anhui province of China. All testicular samples were obtained immediately after the slaughtering process under the veterinary surgical protocol in Fengyang County, Anhui. All pairs of testes were processed by incising the scrotum medially and uncovering the right and left testicles within the tunica vaginalis (Lunstra and Echternkamp, 1982). After removing the fascia tissues, tunica vaginalis, and tunica albuginea from the testis, three slices of testicular samples were cross-cut from the middle aspect of the testis through fine-scale dissection. One cross-cut section was stored in 4% formaldehyde solution for histological assessment, and the others were immediately immersed in liquid nitrogen and stored until total RNA extraction (Wu et al., 2020).

### Histological Assessment

Testicular tissue samples that had been preserved in 4% formaldehyde (Wuhan Servicebio Technology Co., Ltd.) for 72 h were used for histological sections (Fischer et al., 2008). The tissues were sectioned into 6-μm-thick sections and stained with Masson’s trichrome stain. The histomorphology of testicular tissues was assessed using different magnification powers.

### RNA Isolation, Quantification, and Qualification

The testicular samples were sent to a commercial sequencing facility (Beijing Novogene Corporation, China) for Seq-analysis. Total RNA was isolated from the testis samples using the Trizol reagent (Takara, Beijing, China) under sterile conditions and treated with the DNase I enzyme to remove endogenous DNA contamination according to the manufacturer’s protocol. RNA quality and contamination were assessed by 1% agarose gel electrophoresis. The purity was checked using a NanoPhotometer^®^ spectrophotometer (IMPLEN, CA, United States). The total RNA concentration was assessed using a Qubit^®^ RNA Assay Kit in a Qubit^®^ 2.0 fluorometer (Life Technologies, CA, United States). RNA integrity was measured using an RNA Nano 6000 Assay Kit of the Bioanalyzer 2100 system (Agilent Technologies, CA, United States).

### cDNA Library Construction for circRNA Sequencing Analysis

A total of 3 μg of RNA from each testis sample was used to construct the cDNA libraries using the NEB-Next^®^ Ultra-TM RNA Library Prep Kit for Illumina^®^ (NEB, United States). Novogene constructed a chain-specific library by removing ribosomal RNA (Parkhomchuk et al., 2009). First, the ribosomal RNA was removed from the total RNA, and then, the RNA was divided into many short slices of 250–300 bp. The first cDNA strand manufactured from the fragmented RNA was used as the template strand, and random oligonucleotides were used as primers. Subsequently, the RNA strand was degraded with RNase H, and then, the second cDNA strand was manufactured along with dNTPs (dATP, dGTP, dUTP, and dCTP) using the DNA polymerase I system. After purification, the double-stranded cDNA was fixed, followed by the addition of a poly A-tail and sequencing adaptors. A cDNA of approximately 200 bp was separated using the AMPure XP beads. Finally, the USER enzyme was used to degrade the second (uracil-containing) strand of the cDNA, and PCR amplification was performed to obtain the library.

### Alignment of RNA-Sequencing Reads and circRNA Identification

**FIGURE 1:**
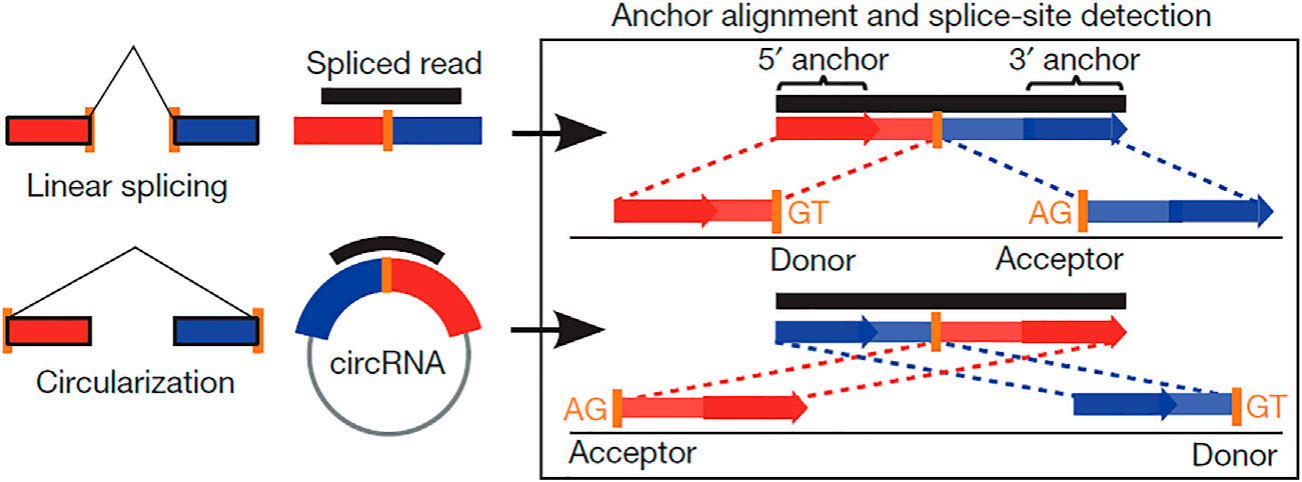
Scheme showing the difference between linear and back-splicing and also illustrating the RNA reads’ accommodation to each other that makes the stable circular RNAs.

To ensure the quality and reliability of the data analysis, it is necessary to filter the original raw data. The low-quality reads (exceeding 50% low-quality bases, e.g., where Q-phred score ≤ 20), reads exceeding 10% possessing (N) nucleotides (where *N*, the proportion of reads that cannot be determined, is greater than 0.002), and reads with seq-adaptors were removed to obtain the clean reads, which were aligned to the ribosomal RNA database using the Bowtie2 tool (Langmead and Salzberg, 2012). The ribosomal RNA-free reads of each calf and bull sample were charted to the reference genome (*Bos taurus*-UMD 3.1.1) by TopHat2 (v. 2.0.3.12) as described previously (Kim et al., 2013). The charted reads of each sample were assembled using String-Tie v1.3.1 (Pertea et al., 2016). Subsequently, 20-mer sequences were removed from both ends of the unmapped reads and aligned according to the reference genomes to identify the exclusive anchor positions within the splicing position. The anchor reads were aligned in the reverse head-to-tail direction that exhibited back splicing (Hansen et al., 2013) and led to key identification of circRNAs (Figure 1). The identified circRNAs were blast for the specific functions against circBase, a database for circRNAs (http://www.circbase.org/) (Glažar et al., 2014), and assigned to different features, such as genomic classification, length distribution, and chromosomal distribution. The circRNAs that did not find their annotations were considered novel.

### Putative Identification and Coding Potential of circRNAs

Putative circRNAs were identified by filtration of anonymous transcripts. To reduce the rates of false positives, the assembled transcripts were filtrated to gain putative circRNAs by the following steps: i) the transcripts having more than one exon were selected, ii) transcripts having more than 200 bp in length and free from exon region overlapping were desired, and iii) transcripts with high expression levels where the FPKM value was more than 2 log2 (fold change) were selected. All known transcripts of the database were analyzed using the Cuffcompare software. The coding potential of circRNAs was determined using the software programs Coding–Non-Coding Index (CNCI) (Sun et al., 2013), Pfam-scan (Bateman et al., 2004; Finn et al., 2014), and coding potential calculator (CPC) (Kong et al., 2007). All transcripts identified with coding potential were refined, and those without coding potential were recognized as putative circRNAs.

### Expression Pattern of Differentially Expressed circRNAs and miRNA Endogenous Sponge

The circRNA gene expression level was assessed using the fragments per kilobase of transcript sequence per million base pairs sequenced (FPKM) value. The significance of DE circRNAs at the gene or transcript level was analyzed, and functional genes relevant to the developmental groups were identified. Cuffdiff (v2.1.1) software was used to project the TPM levels of circRNAs (Trapnell et al., 2010). An edgeR kit (http://www.rproject.org/) was used to classify differentially expressed circRNAs through samples. CircRNAs with a fold change greater than or equal to 2 and a *p*-value *<* 0.05 in comparison between the samples are differentially expressed. We identified the endogenous sponge interaction between the differentially expressed circRNA and miRNA by means of three software programs: miRanda (v. 3.3a), MIREAP, and TargetScan (v. 7.0).

### Bioinformatics Analysis of GO and KEGG

The GO term analysis and classification provides significant source genes that are involved in different biological functions, and DE circRNA genes were employed by the GO-seq R package (Young et al., 2010). All source genes in the pathways and the background genes were charted to the GO terms in the database (http://www.geneontology.org/). The GO terms with the corrected p-value (*p <* 0.05) were recognized as relatively enriched by DE genes according to the definition of the hyper-geometric test. The significance analysis of the term enrichment was corrected by FDR, and the corrected *p*-value (Q-value) was obtained (Ashburner et al., 2000). The KEGG pathway-based study further elaborated the explanation of source genes and their biological functions (http://www.genome.jp). KOBAS software was used to test the enriched DE source genes in the KEGG pathways, as suggested by (Mao et al., 2005). The enrichment analysis significantly identified the signaling and metabolic pathways, to which circRNA source genes and total background genes were charted.

### Validation of the circRNAs Gene *via* RT-qPCR

Whole RNA was extracted from immature and mature groups using the Trizol reagent (Life Technologies, 182805, United States), and its concentration was measured using a Nanodrop spectrophotometer. The good-quality RNAs were then converted into cDNAs using a QuantiTect Reverse Transcription Kit (Qiagen, 205311, Germany) in accordance with the manufacturer’s protocol. The software programs Primer 3 web version 4.0.0 and basic local alignment search tool (BLAST; https://blast.ncbi.nlm.nih) were used to design gene-specific primers. The obtained PCR products were detected by 3% agarose gel and Sanger sequencing (Sangon Biotech, Shanghai, China). The divergent primers were used in this study and are presented in Supplementary File 1. The expression of circRNAs was detected by using a StepOnePlus Real-Time PCR System (Applied Biosystems, United States) using the FastStart SYBR Green Master Mix (Roche, Germany). Each PCR comprised 7.5 μl 2× SYBR Green PCR Master Mix, 1.5 μl cDNA, 0.5 μl each of reverse and forward primers, 4.7 μl nuclease-free water, and 10.3 μl ROX dye. The following q-PCR thermal cycles were carried out: i) predenaturation at 95°C for 10 min, ii) followed by 45 denaturation amplification cycles at 95°C for 15 s, iii) annealing at 60°C for 10 s, and iv) an extension cycle at 72°C for 20 s. The quantification cycle (Cq) of each target gene was normalized to that of the reference *GAPDH* gene. The q-PCR experiment was performed in triplicate to minimize the risk of error in the experiments. The Cq values were obtained and transferred to a Microsoft Excel sheet for further relative quantification analysis using the 2−ΔΔCt method (Sherman and Lempicki, 2009).

### Statistical Analysis

Data on circRNA transcripts of interest from immature and mature testicular tissues were analyzed using the student *t*-test (SPSS 17.0) and presented as mean ± SEM. Before conducting the *t*-test, the data distribution and variances between the two groups were evaluated and found to be normal and homogeneous, respectively. Mean values were believed to be significantly different with *p < 0.05 and **p < 0.01.

## Results

### Histomorphology of Testes

**FIGURE 2:**
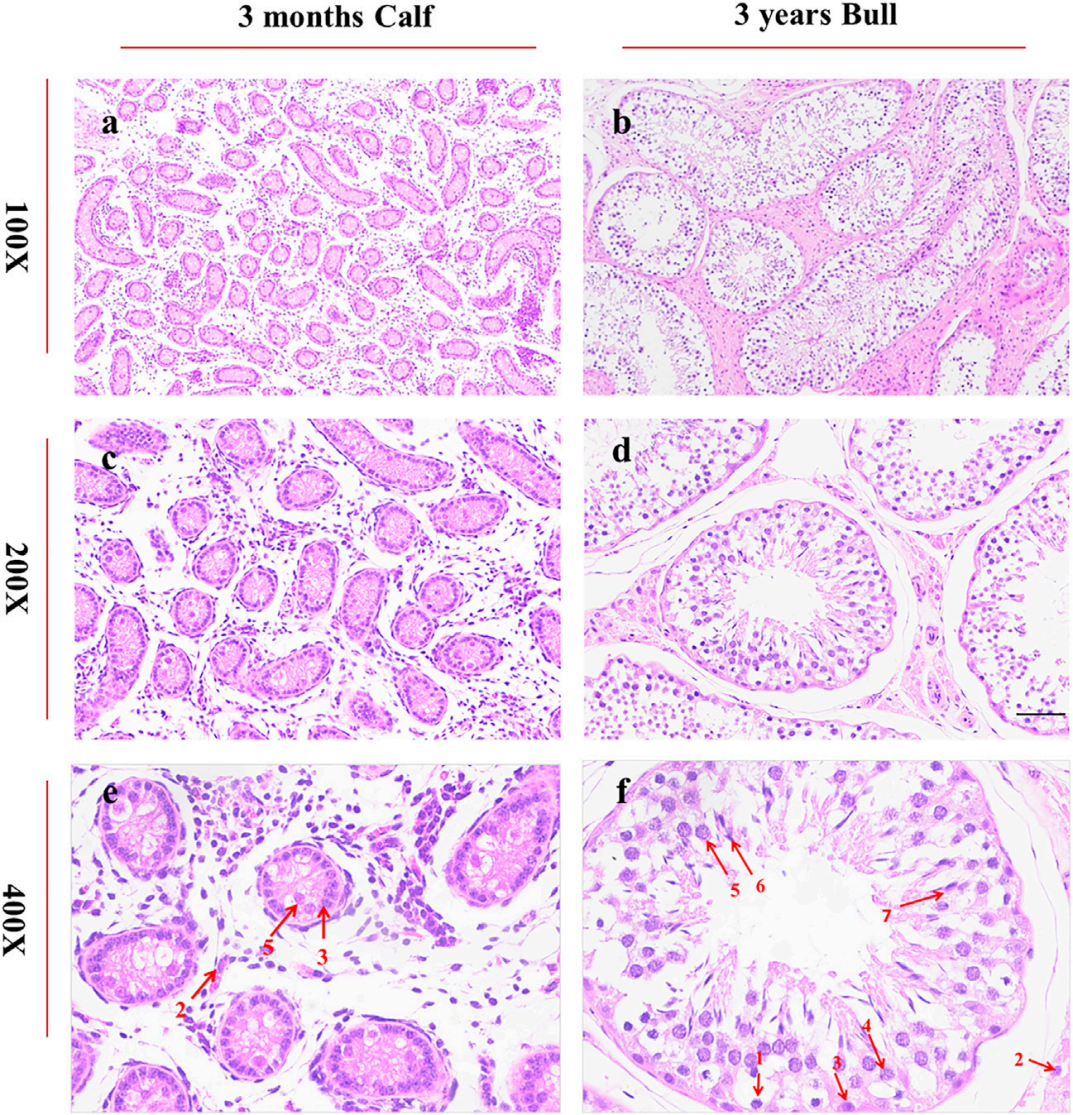
Histomorphological analysis of bull testicular tissues at ages of 3 months and 3 years was performed under a microscope at ×100, × 200, and × 400 magnifications. The segments **(A,C,E)** represent the morphology of 3-month-old testes, whereas **(B,D,F)** represents the morphology of 3-year-old bulls. The red arrows indicate different cell types. 1: spermatogonia, 2: Leydig cells, 3: Sertoli cells, 4: spermatocytes, 5: round spermatids, 6: elongated spermatids, and 7: sperms.

A histological study of bull (*B. taurus*) testes showed a noteworthy difference between immature and mature stages. Under 100X microscopic examination, the diameter of seminiferous tubules was much smaller in immature than in mature testes. At the same time, similar conditions were observed in the interstitial connective tissue of immature and mature testes, as highlighted in Figures 2A,B. Under 400X microscopic examination, we obtained further details and observed more developmental stages of biological spermatozoa in mature than in immature testes. Sertoli cells were significantly increased in number and were found very near the basement membrane of seminiferous tubules in mature testes, as shown in Figures 2E,F.

### Transcriptome Sequencing of circRNAs in Immature and Mature Bull Testes

**FIGURE 3:**
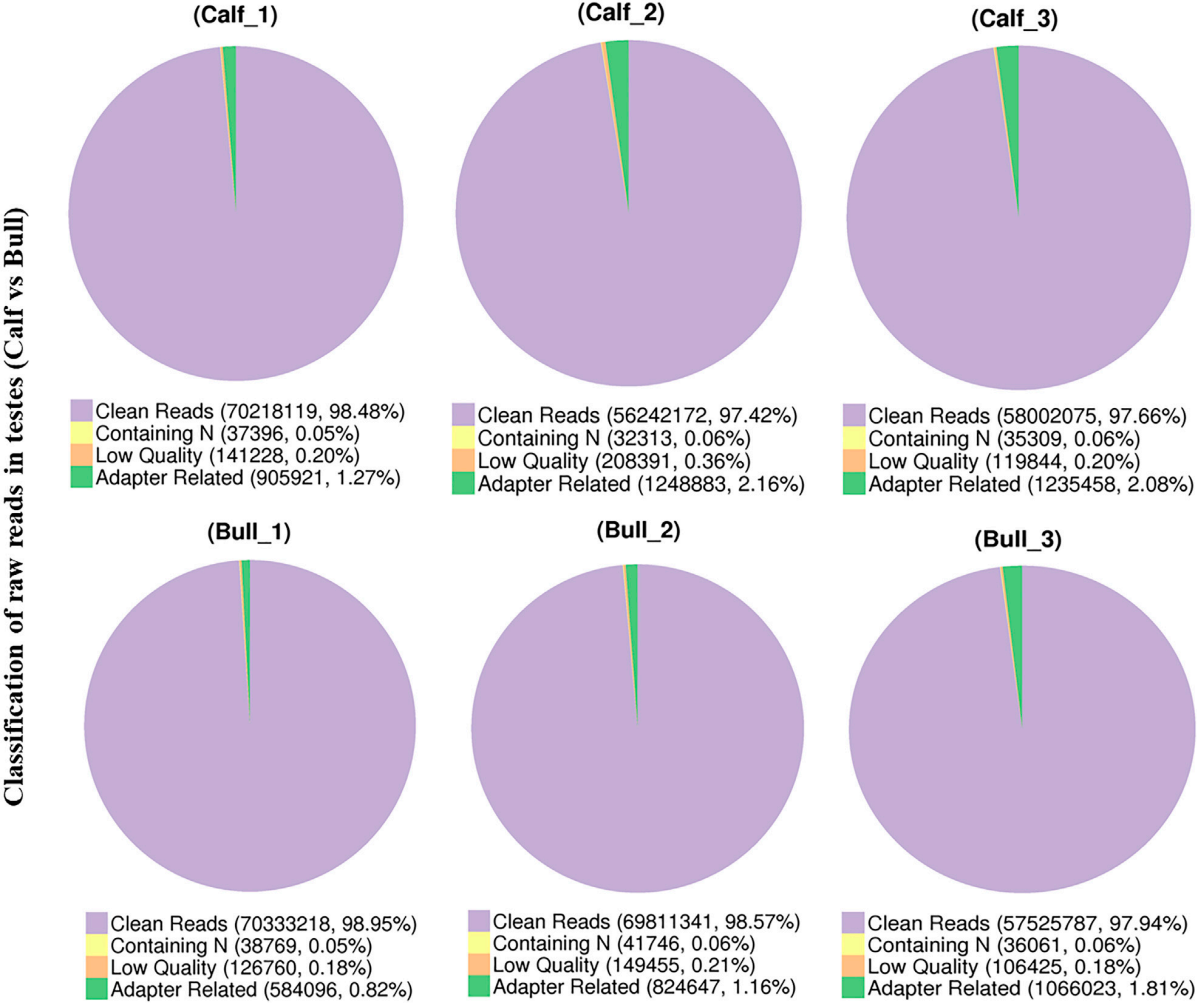
Pie charts show the raw reads of sequencing data from immature and mature bull testis samples that were subjected to RNA sequencing analysis.

A total of six experimental animals, three immature (3 ± 0.014 months) and three mature (3 ± 0.24 years), were selected from a local cattle station, and their testis samples were collected. To investigate the circRNA profiles of the immature and mature testis tissues, we prepared the RNA-seq libraries of all tissue samples. The raw reads of sequencing data were acquired using an Illumina HiSeq 4000 platform (Illumina, Inc, San Diego, CA, United States). Raw reads were filtered and classified into different segments, including clean reads (70,333,218, 98.95%), N-containing reads (38,769, 0.05%), low-quality reads (126,760, 0.18%), and adopter-related reads (584,096, 0.82%) (Figure 3). The raw reads for each sample ranged between 118,785,372 and 142,165,686, while the clean reads per sample ranged between 116,004,150 and 140,666,436. Thus, the raw and clean reads together yielded 90 GB of data, and the GC content ranged from 47.09 to 53.57% (Supplementary File 2). All Q20 values of the subjected reads in the six samples exceeded 97%, as shown in Supplementary File 3. More than 95.5% of the clean reads were coordinated to *B. taurus* UMD3.1.

### Correlation and Differential Analysis Between the Testis Samples

**FIGURE 4:**
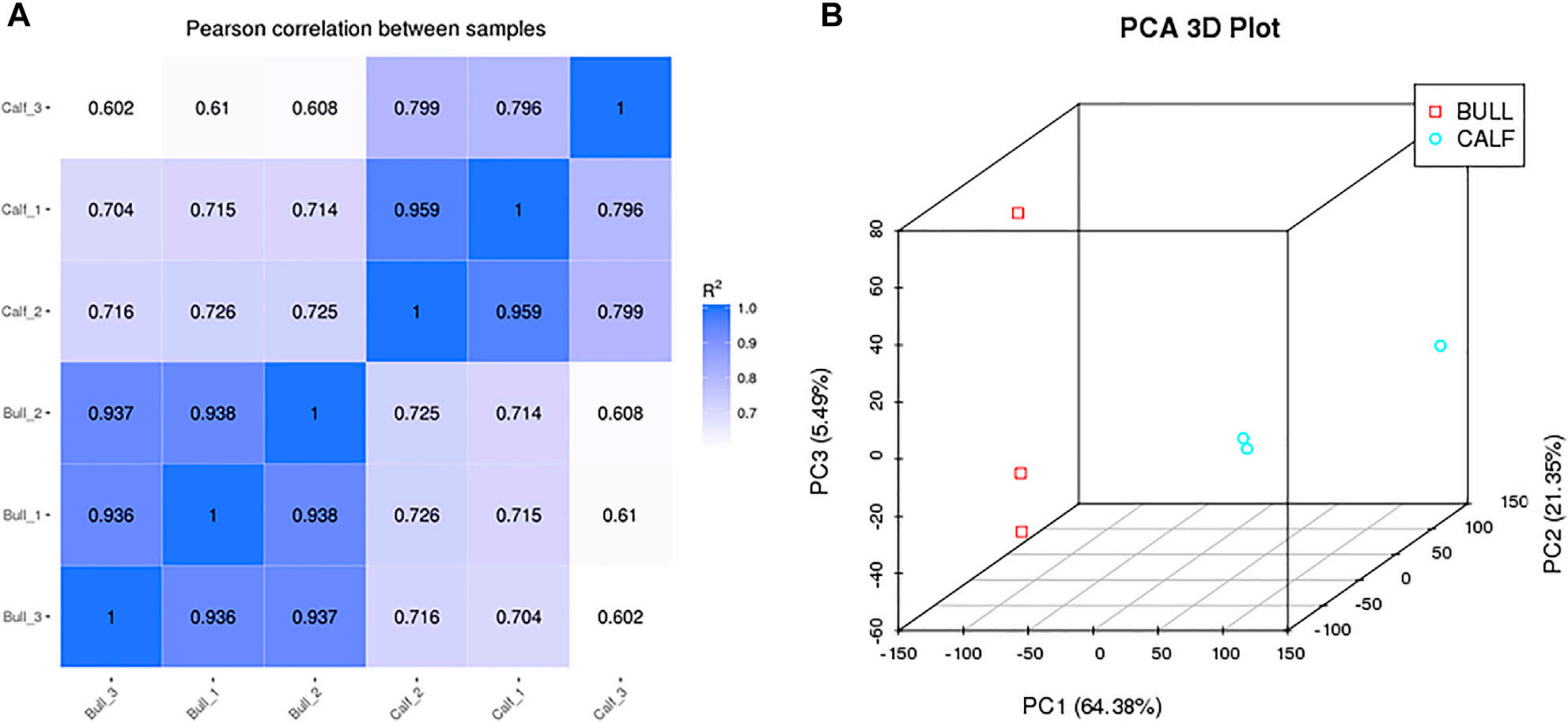
Pearson correlation and principle component analysis were constructed between groups and samples. **(A)** Correlation among the sequencing samples of calf and bull testes. **(B)** PCA 3D plot among the sequencing samples.

The testis samples were collected from three calves (Calf1, Calf2, and Calf3) and three bulls (Bull1, Bull2, and Bull3). The Pearson’s correlation heatmap was constructed according to the expression profiles for samples’ quality control. All samples in the calf and bull groups showed the linear correlation (Figure 4A). The differences of transcripts of two groups demonstrated in the sample are shown in the PCA 3D map (Figure 4B). We also discovered that bull and calf groups have noticeable differences, whereas the testis samples of calves and bulls clustered separately and showed the difference.

### Identification and Characterization of circRNAs

**FIGURE 5:**
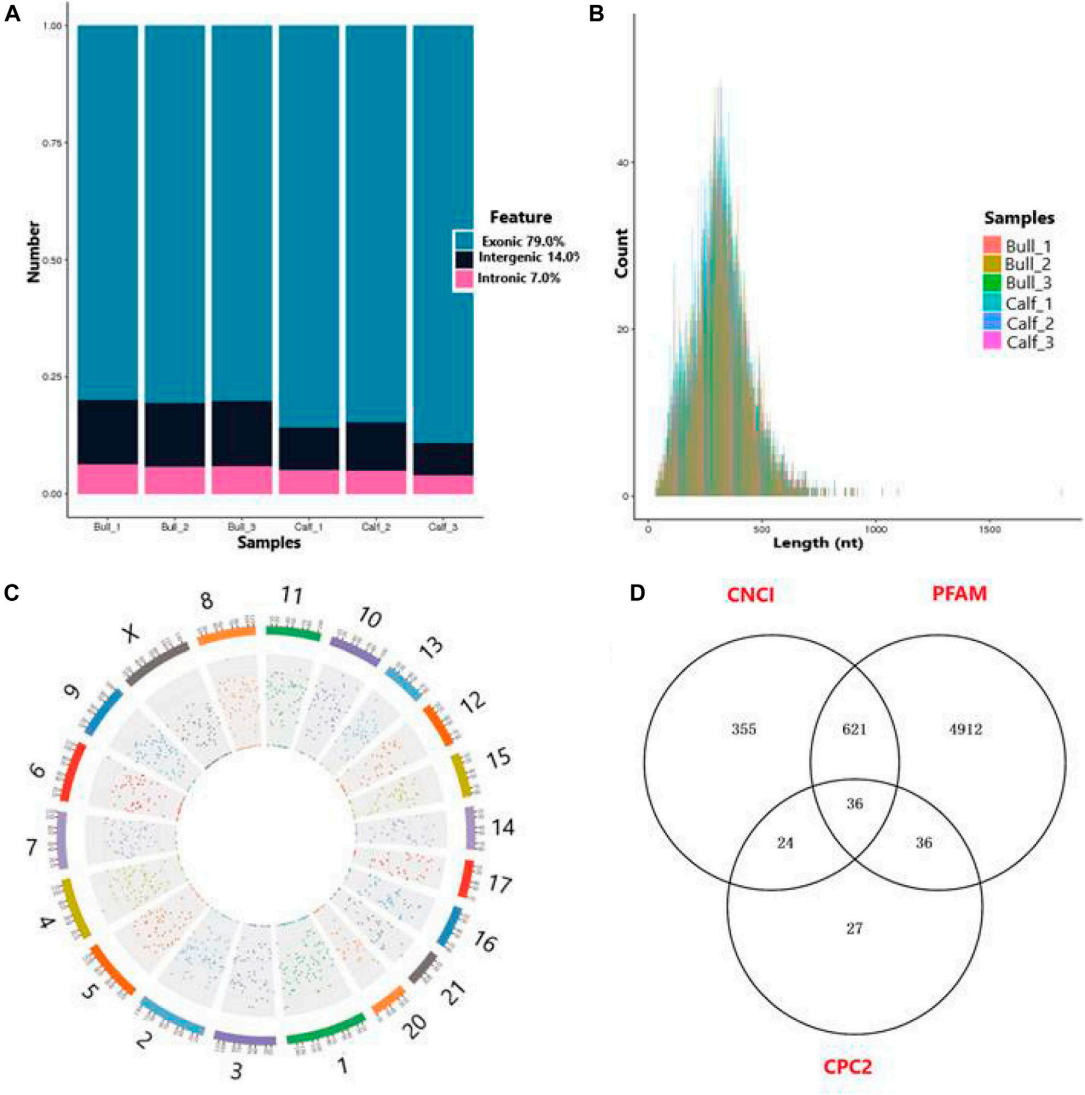
General features of circRNAs in immature and mature bull testes. **(A)** Distribution of circRNAs in the genomic region and high magnitude identified from exon, intergenic, and intron parts of the genome. **(B)** Length distribution of circRNAs, while colors represent the different lengths of circRNAs in calf and bull testis samples. **(C)** Distribution of testis circRNAs on the cattle chromosomal sets. **(D)** Venn diagram shows the coding potential results of circRNAs and intersection of coding tools such as coding–non-coding index (CNCI), PFAM, and coding potential calculator (CPC2).

A total of 17,013 putative circRNAs were recognized with at least one read spanning and head-to-tail splicing in immature and mature testes. Based on their genomic location and features, circRNAs were divided into three subclasses, denoted as circ-exon (79.0%), circ-intergenic (14.0%), and circ-intron (7.0%) (Figure 5A). The total length plot quantile of circRNAs (approximately 75%) was not more than 1,000 nt, and the average length was 400 nt, as shown in Figure 5B. According to their host gene site, all the circRNAs were widely distributed on all sets of chromosomes, while none of the circRNA was mapped to the mitochondrial genome. The total available set of chromosomes generated 100 circRNAs in both developmental stages of the *Bovine* testis (Figure 5C). The coding potential of circRNAs was analyzed using the software programs CPC2, CNCI, and PFAM, and 6,011 coding circRNAs were identified in all samples (Figure 5D). Detailed information on the top 40 up- and downcoding circRNAs is listed in Table 1.

**TABLE 1:** Top 40 regulated coding circular RNAs in immature and mature testicular tissues of Wandong cattle.

Novel circRNA Id	Chromosome	Total cds length	Classification	Source gene	Fold change	*P*_*adj*_ value
Top 20 novel upregulated coding circRNAs						
novel_circ_0024958	Chr 4	608	Exonic	*KMT2E*	3.2126	0.008797
novel_circ_0001818	Chr 11	325	Exonic	*EHMTI*	7.1764	0.014192
novel_circ_0027596	Chr 6	308	Exonic	*NSD2*	7.4895	0.009261
novel_circ_0006800	Chr 15	596	Exonic	*ATM*	2.0689	0.028979
novel_circ_0005451	Chr 13	402	Exonic	*RBLI*	3.0064	0.014784
novel_circ_0012012935	Chr 1	397	Exonic	*GSK3B*	3.206	0.026266
novel_circ_0029602	Chr 7	477	Exonic	*CDC25C*	4.0429	0.001681
novel_circ_0026085	Chr 5	506	Exonic	*SMCIB*	4.375	0.013226
novel_circ_0024710	Chr 4	402	Exonic	*DBF4*	3.816	9.32E-07
novel_circ_0003626	Chr 12	451	Exonic	*CCNAI*	8.2913	0.001968
novel_circ_0031633	Chr 9	436	Exonic	*AFDN*	2.4391	0.04536
novel_circ_0004558	Chr 13	630	Exonic	*PARD3*	2.8041	0.007949
novel_circ_0006892	Chr 15	594	Exonic	*CADMI*	7.4286	0.009651
novel_circ_0017085	Chr 24	546	Exonic	*LAMA3*	7.0164	0.07625
novel_circ_0009598	Chr 17	359	Exonic	*PXN*	7.1673	0.014472
novel_circ_0015698	Chr 22	431	Exonic	*SUCLG2*	4.4091	0.024862
novel_circ_0018225	Chr 25	270	Exonic	*ABAT*	6.456	0.038294
novel_circ_0029184	Chr 7	307	Exonic	*TJP3*	6.9245	0.020237
novel_circ_0031633	Chr 9	436	Exonic	*AFDN*	2.4391	0.04536
novel_circ_0024833	Chr 4	592	Exonic	*HGF*	7.3916	0.010582
Top 20 novel downregulated coding circRNAs						
novel_circ_0025557	Chr 4	655	Exonic	*AASS*	−4.2615	0.04536
novel_circ_0023110	Chr 3	272	Exonic	*VAV3*	−7.4813	0.010743
novel_circ_0024329	Chr 5	455	Exonic	*KMT2C*	−3.0993	0.031917
novel_circ_0033406	Chr X	399	Exonic	*TMLHE*	−3.0993	0.016812
novel_circ_0012012847	Chr 1	906	Exonic	*NECTINN3*	−2.9046	0.016725
novel_circ_0012012395	Chr 1	561	Exonic	*NCAM2*	−4.3218	0.009566
novel_circ_0023110	Chr 3	272	Exonic	*VAV3*	−7.4813	0.010743
novel_circ_0000398	Chr 10	607	Exonic	*NEOI*	−3.4837	0.031452
novel_circ_0007021	Chr 15	298	Exonic	*RRAS2*	−2.5529	0.001203
novel_circ_0031262	Chr 8	618	Exonic	*GKAPI*	−9.4111	0.000179
novel_circ_0022366	Chr 2	305	Exonic	*UNC80*	−5.385	0.004455
novel_circ_0032345	Chr 9	368	Exonic	*ZNF292*	−2.1687	0.00742
novel_circ_0024463	Chr 4	546	Exonic	*DYNCIII*	−4.7909	0.007542
novel_circ_001012674	Chr 1	319	Exonic	*ST3GAL6*	−3.4838	0.007765
novel_circ_0012012395	Chr 1	561	Exonic	*NCAM2*	−4.3218	0.009566
novel_circ_0028513	Chr 6	164	Exonic	*EPHA5*	−7.5188	0.00975
novel_circ_0031677	Chr 9	696	Exonic	*RIMSI*	−3.1598	0.010582
novel_circ_0020402	Chr 29	187	Exonic	*MYRF*	−7.506	0.010879
novel_circ_0014496	Chr 21	264	Exonic	*SH3GL3*	−3.4807	0.002134
novel_circ_0026164	Chr 5	468	Exonic	*TMTC2*	−3.8141	0.012555

### Differentially Expressed Gene and circRNAs in Testicular Tissues

**FIGURE 6:**
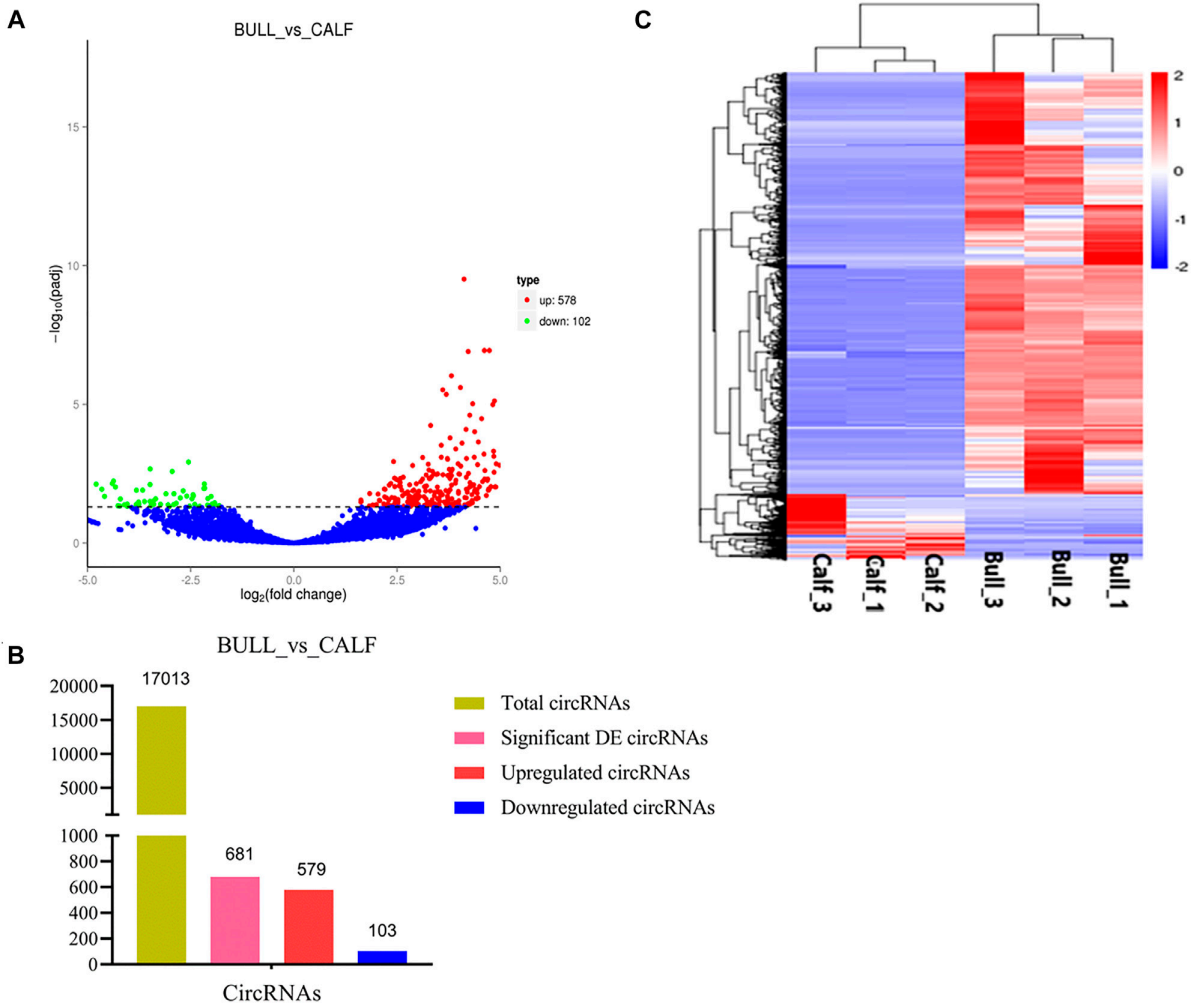
Expression patterns of circular RNAs (circRNAs) and protein-coding genes. **(A)** Volcanic plots represent log10 (*p*-value) vs log2 fold difference in circRNAs in the fragments per kilobase of transcript per million mapped reads (FPKM) values between immature and mature testes. **(B)** Total abundance of circRNAs and differentially expressed (DE) highlights between calf vs bull. **(C)** Heatmap of DE circRNAs among calves and bulls. Red indicates upregulated coding genes, and blue indicates downregulated gene products.

After the quantification process, the expression pattern of circRNAs was identified using Cuffdiff and Ballgown tools. A total of 681 DE circRNAs at the gene level were detected in immature and mature stages. The expression levels of circRNAs in the testis samples were calculated, taking into account the parameter of significance, whereas the log2 fold change was considered higher than or equal to 2 and *p*-adjusted < 0.05. According to the significance criteria, we detected 578 upregulated and 102 downregulated circRNAs between bull and calf testis samples. These up and down highlights of DE genes are displayed in volcanic plots (Figures 6A,B), and the list of total and DE circRNAs is shown in Supplementary Files 4–6. We also analyzed the DE circRNA by the hierarchical clustering method, which is another way to display DE genes and to cluster all the genes with similar expression patterns that may have common functions in metabolic and signaling pathways. The gene clusters on the left side were formed because of similar expression patterns (fold change >2, *p <* 0.05); the columns presented calves and bulls, and the expression from blue to red was gradually upregulated (Figure 6C).

### GO and Enriched KEGG Pathways Analysis

**FIGURE 7:**
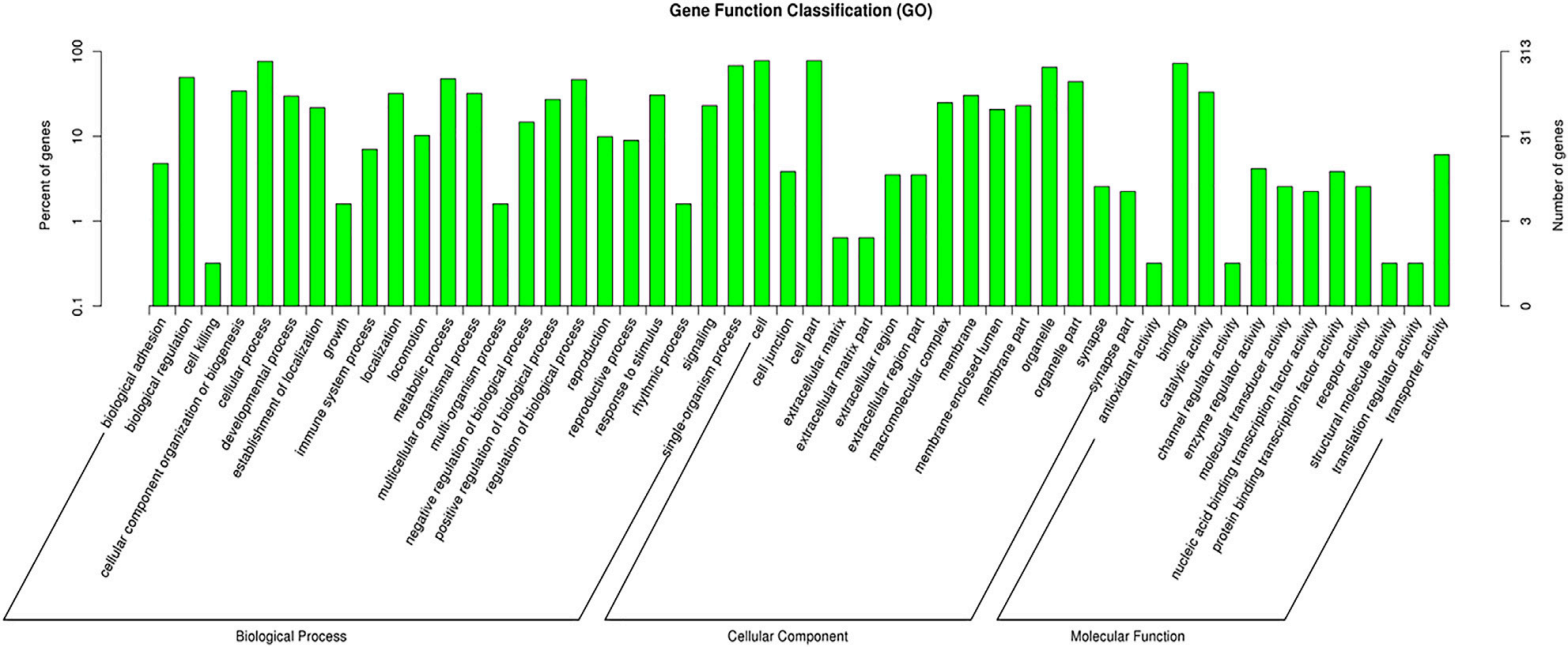
GO enrichment analysis of differentially expressed (DE) circular RNA (circRNA) genes between immature and mature testicles was performed. The DE circRNA genes are divided into the following three functional categories: biological processes (BP), cellular components (CC), and molecular functions (MF), while the left and right *y*-axes show the percentage and numbers of circRNAs host genes, respectively.

**FIGURE 8:**
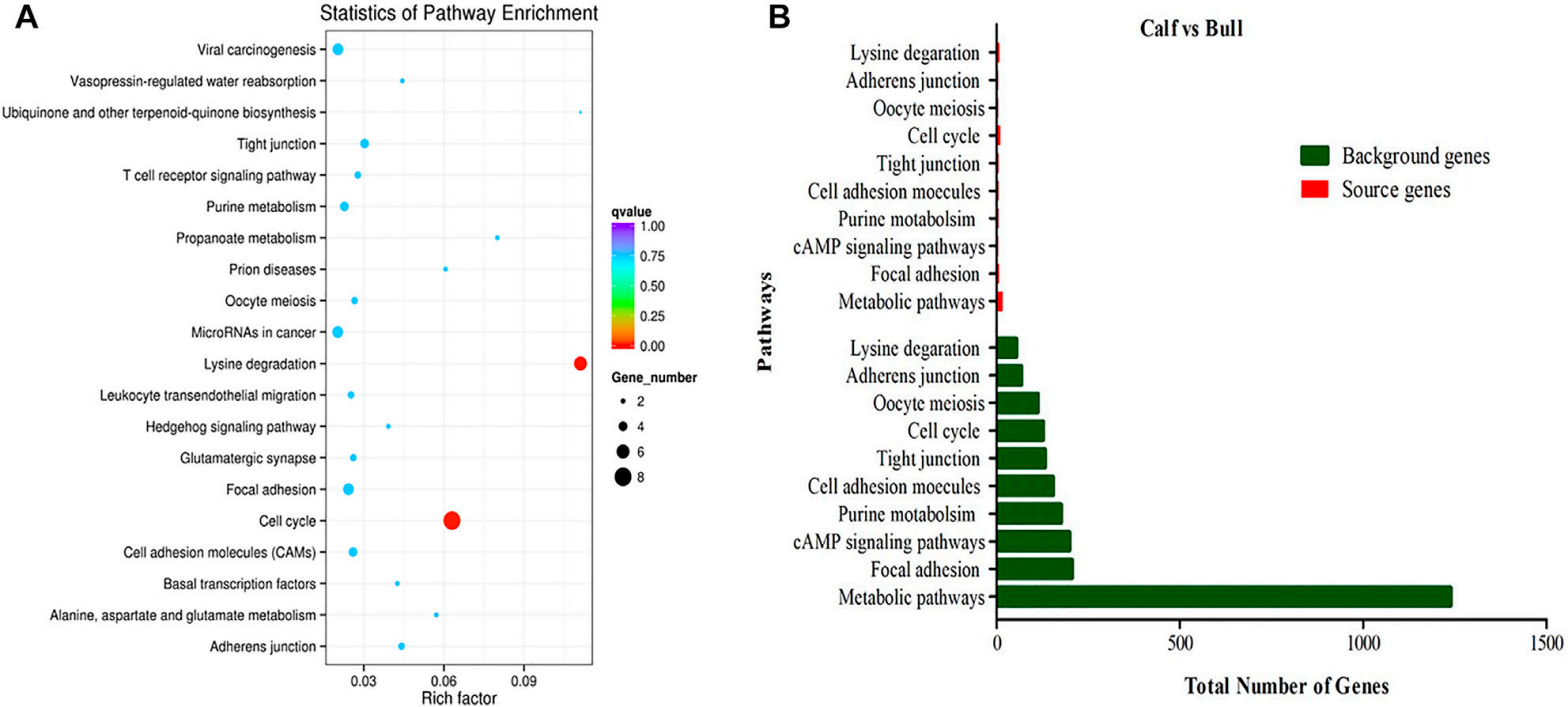
KEGG enrichment pathway analysis was performed. **(A)** Top 20 enriched pathways of differentially expressed (DE) circRNA host genes in immature and mature stages of testicular growth. The color dots represent different signaling pathways that are regulated by the circRNA host genes. The size and different colors of dots show the numbers of genes and significance levels in enriched pathways. **(B)** Top 10 most relevant reproduction pathways representing the total background genes and significant source genes of circRNAs.

CircRNAs are closed-loop structures that are most commonly generated by back-splicing events between the exons of coding genes. In a limited way, circRNAs regulate the host gene and thus perform the desired function at the genetic level. Hence, the analysis of GO terms and circRNA source gene functions might provide new insights. Many GO classification terms were significantly enriched in the source genes of circRNAs. A list of 26,815 source genes significantly associated with DE circRNAs was submitted to the database for functional annotation and GO classification analysis, resulting in the identification of 50 significant GO terms, as shown in Figure 7. The functional annotation study revealed that several source genes actively participate in spermatogenesis, sperm morphology, developmental stages, and reproduction. The functional classification of source genes showed that their products were assigned to the three GO categories of biological processes, cellular components, and molecular functions (Supplementary File 7). To further understand the role of circRNA host genes in the developmental stages of the *Bovine*, a pathway analysis was applied using the KEGG pathway database and a total of 147 signaling pathways linked to circRNA gene products were identified (Supplementary File 8). The top 20 statistically enriched pathways (*p <* 0.05) were subjected to further analysis and were found to be significantly associated with signaling pathways such as lysine degradation, cell cycle, propanoate metabolism, adherens junction, and cell adhesion molecules (Figure 8). The host genes such as *KMT2E*, *EHMT1*, and *NSD2* were mediated by the lysine degradation signaling pathways but actively participated in the reproductive traits of the *Bovine*. Similarly, *ATM*, *GSK3B*, and *CCNA1* are host genes of the cell cycle signaling pathway and influence the biological descriptions relevant to spermatogenesis and testis development. The most significantly enriched pathways and DEGs are listed in Table 2.

**TABLE 2:** Most enriched biological pathway terms and regulated DE genes in immature and mature testes.

Pathway terms	Rich factor	q-value	Gene number	Gene name
Lysine degradation	0.111111111	0.006486719	6	*AASS,KMT2C,KMT2E,EHMT1,TMLHE, NSD2*
Cell cycle	0.062992126	0.007955094	8	*ATM,RBL1,GSK3B,CDC25C, SMC1B, DBF4,CCNA1*
Propanoate metabolism	0.08	0.753456013	2	*SUCLG2,ABAT*
Adherens junction	0.044117647	0.753456013	3	*NECTIN3,AFDN,PARD3*
Prion diseases	0.060606061	0.753456013	2	*NCAM2,C5*
Tight junction	0.03030303	0.753456013	4	*TJP3,RRAS2,AFDN,PARD3*
Alanine, aspartate, and glutamate metabolism	0.057142857	0.753456013	2	*GLS,ABAT*
Focal adhesion	0.024271845	0.753456013	5	*VAV3,LAMA3 LAMA3, GSK3B, PXN*
Vasopressin-regulated water reabsorption	0.044444444	0.753456013	2	*AQP4,DYNC1I1*
Cell adhesion molecules (CAMs)	0.025974026	0.753456013	4	*NECTIN3,NCAM2,NEO1,CADM1*
Basal transcription factors	0.042553191	0.753456013	2	*GTF2A1,TAF2*
Hedgehog signaling pathway	0.039215686	0.753456013	2	*GSK3B,CSNK1G3*
T-cell receptor signaling pathway	0.027777778	0.753456013	3	*GSK3B, DLG1, VAV3*
Oocyte meiosis	0.026548673	0.753456013	3	*SMC1B, CDC25C, FBX O 43*
Purine metabolism	0.022727273	0.753456013	4	*PDE10A, FHIT, PRIM2,AK8*
Glutamatergic synapse	0.026086957	0.753456013	3	*GLS, HOMER1, DLGAP1*
Viral carcinogenesis	0.020325203	0.753456013	5	*DLG1, CCNA1, GTF2A1, PXN, RBL1*
MicroRNAs in cancer	0.020242915	0.753456013	5	*ZFPM2, ATM, GLS, CDC25C, EZH2*
Leukocyte transendothelial migration	0.025210084	0.753456013	3	*VAV3, AFDN, PXN*

### Prediction of Deferentially Expressed circRNA and miRNA Endogenous Sponges

**FIGURE 9:**
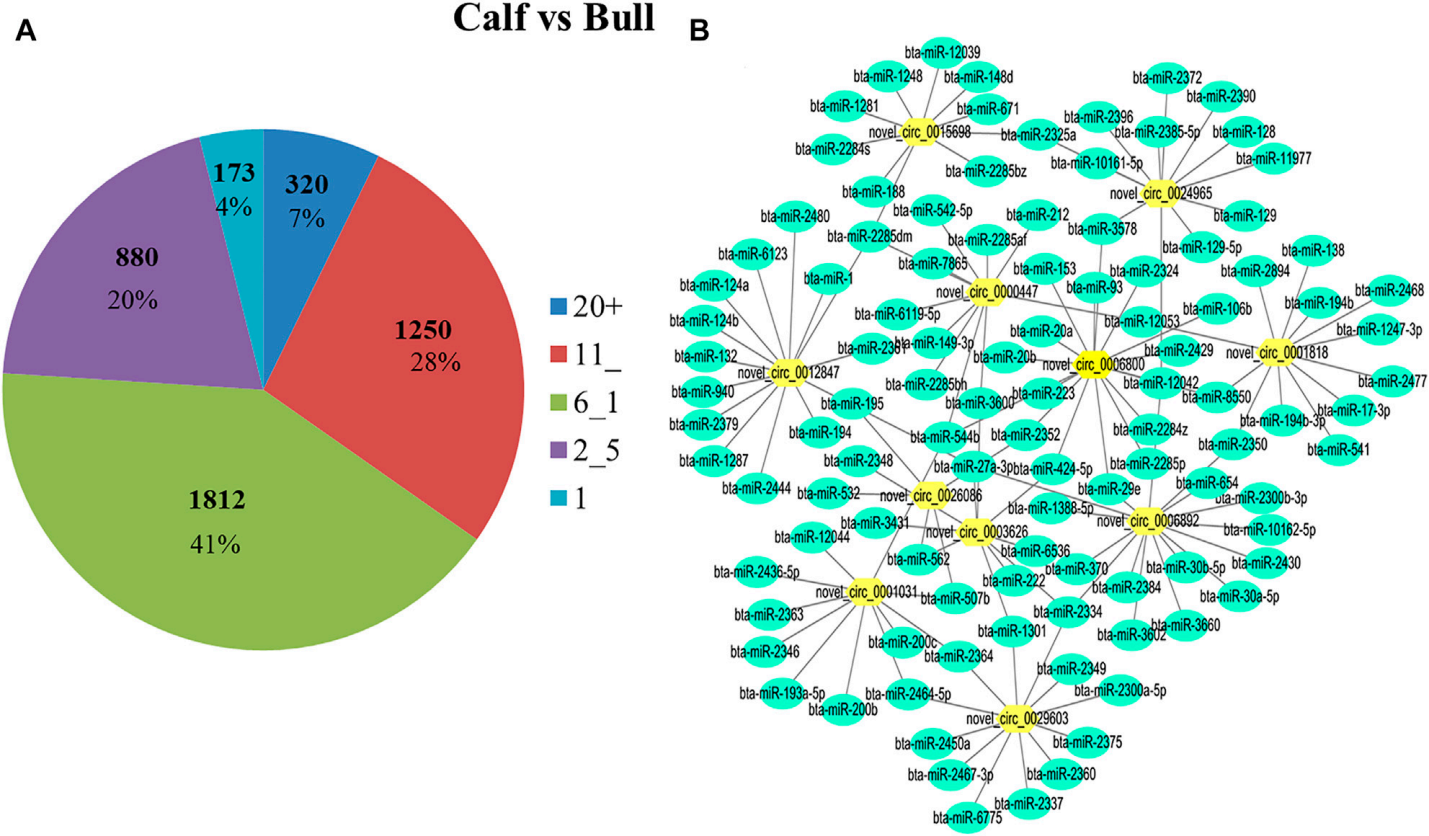
Differentially expressed (DE) circRNA and miRNA networks in the bull testis were identified. **(A)** During binding interaction, different numbers of miRNA targets were processed by circRNAs. **(B)** DE circRNAs and the predicted targeted miRNAs network were identified for calf and bull testes. Green nodes represent the targeted miRNAs, and yellow nodes show circRNAs.

Some studies in recent years have suggested that miRNAs play a role in all aspects of spermatogenesis and testis formation; thus, miRNAs could be used as biomarkers for reproductive traits (Yadav and Kotaja, 2014). Reports also suggested that circRNAs act as miRNA sponges and play an important role in regulating gene expression in posttranscriptional processes (Kulcheski et al., 2016). We analyzed further to determine whether circRNAs found in immature and mature testes act as endogenous miRNA sponges. We noticed that a total of 4,300 circRNAs have potential binding miRNA sites, but other circRNAs were predicted to have no possible binding targets for miRNAs. Each circRNA may attach to one or more targeted miRNAs. As a result, the percentage of circRNAs consisting of different numbers of miRNA targets was further tested. The majority of circRNAs have at least two miRNA-binding sites; here, the proportion of circRNAs containing 6–10 miRNA targets is the largest, as illustrated in Figure 9A. The binding interactions between selected DE circRNAs in testicular tissues and their predicted miRNA targets are shown in Figure 9B. Recent studies have proposed that circRNAs act as miRNA sponges and play a critical role in regulating gene expression in pathways through complex (circRNAs–miRNAs genes) chains (Hansen et al., 2013; Memczak et al., 2013). Approximately 758 miRNAs have been predicted, and many of them, such as bta-miR-204, bta-miR-532, and bta-miR-34 groups, have been correlated with spermatogenesis (Zhang et al., 2015).

### Validation of DE circRNA-Seq Results by RT-qPCR

**FIGURE 10:**
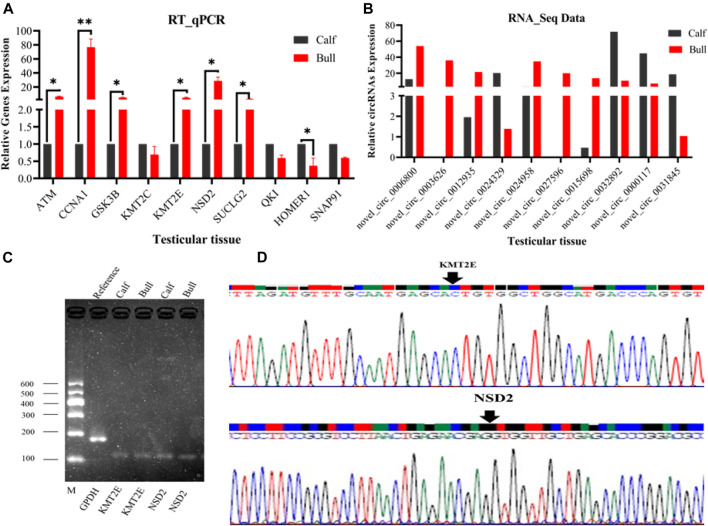
Confirmation of the differentially expressed (DE) circRNAs was done by RT-qPCR at two different developmental stages of the *Bovine* testis. The figures **(A,B)** show the expression patterns of ten DE circRNAs and their host genes (*ATM, CCNA1, GSK3B, KMT2C, KMT2E, NSD2, SUCLG2, QKI, HOMER1*, and *SNAP91*) in the immature and mature groups. The data were measured by the 2−ΔΔCt method, and GAPDH was used as a housekeeping gene. The data are presented as the mean ± SEM, *p < 0.05, **p < 0.01. **(C)** PCR products of circRNA were confirmed *via* gel electrophoresis, where M represents marker I. **(D)** Back-splice junction of circRNA was confirmed by the Sanger sequencing analysis.

To justify the sequencing analysis of DE circRNAs through RT-qPCR, we selected the truly significant and exonic DE circRNAs for validation. We randomly selected nine circRNAs of different nucleotide richness values and lengths, and divergent primers were prepared to verify the expression levels of circRNAs in the testis. Ten host genes were proposed for circRNA detection, of which seven, including ATM serine/threonine kinase (*ATM*), cyclin A1 (*CCNA1*), glycogen synthase kinase 3 beta (*GSK3B*), lysine methyltransferase 2C (*KMT2C*), lysine methyltransferase 2E (*KMT2E*), nuclear receptor binding SET domain protein 2 (*NSD2*), and succinate-CoA ligase GDP-forming subunit beta (*SUCLG2*), were upregulated. The remaining three genes, including QKI, KH domain-containing RNA binding (*QKI*), homer scaffold protein 1 (*HOMER1*), and synaptosome-associated protein 91 (*SNAP91*), were downregulated. The RT-qPCR analysis validated the expression of all nine circRNAs, which were found to regulate the functions of the aforementioned genes, as shown in Figures 10A,B. The gel electrophoresis findings showed that the PCR products of circRNA were single bands, which designated the presence of specific circRNAs, Figure 10C. Additionally, the back-splice junction of circRNAs was highlighted by further Sanger sequencing of the PCR products, Figure 10D. These genes work in specific reproductive-related pathways, such as lysine degradation, cell adhesion molecules (CAMs), focal adhesion, cell cycles, and adherens junctions, thus supporting reproductive functions in animals. Therefore, the obtained results confirmed the accuracy of the ribo-depleted RNA-seq results.

## Discussion

Mammalian testicular growth and spermatogenesis are complex biological transformation processes that are regulated by a strong combination of coding and noncoding genes and transcriptomes (Card et al., 2013; Djureinovic et al., 2014). Testicular growth includes the growth of testicular tissues during the embryonic and postnatal phases (Svingen and Koopman, 2013). The bull testes first gradually expand until approximately 25 weeks of growth and then rapidly expand until adolescence at 37–50 weeks of age (Rawlings et al., 2008). To analyze the molecular biology of testis development and reproductive transformation in *Bovine* species, we selected *n* = 3 animals of two different age groups: 3-month-old (immature) calves and 3-year-old (adult) bulls of a local Wandong cattle breed. Gao et al. (2018) tested the similar experimental design in the Qinchuan cattle testis and selected *n* = 1 animals of two different age groups: neonatal (1 week) and mature bulls (4 years).

Previously, nc-RNAs were regarded as useless stocks or junk RNAs. However, various types of ncRNAs, such as miRNAs, PIWI-interacting RNAs (pi-RNAs), long noncoding RNAs (lnc-RNAs), and circRNAs (Lee et al., 1993; Girard et al., 2006; Memczak et al., 2013; Ulitsky and Bartel, 2013), have been identified and reported to play important roles in cellular and tissue development. In recent years, research has focused on the functional features of nc-RNAs, and much work has been reported specifically in animal reproduction (Svingen and Koopman, 2013). In addition, lnc-RNAs play a regulatory role in the testis and are expressed more strongly in mammalian testes than in other organs (Soumillon et al., 2013). While circRNAs are essential members of the nc-RNA family, they are also known to be involved in animal reproduction and disease control (Wang et al., 2016). Limited studies have characterized circRNAs in reproductive organs, such as placentae, embryos, and testes, and in cell types including oocytes, granulosa cells, immature sperm cells, seminal plasma cells, and mature sperm cells (Dong et al., 2016; Quan and Li, 2018; Chioccarelli et al., 2019). circRNAs were identified between the good and poor sperm subpopulations in terms of kinetic parameters and morphological characteristics. These comparative data highlighted 148 DE circRNAs between the two semen quality classes (Dong et al., 2016). Previous studies have reported that circRNAs are the primary regulators of multiple biological processes, but they have not been methodically explored in the *Bovine* testis during reproductive development. In this study, we identified and marked circRNAs in *Bovine* testes and discovered possible genes that determine the development of testes.

A total of 17,013 and 681 coding circRNAs were identified in the *Bovine* testes at two different immature and mature stages. You et al. (2015) reported that the second highest number of circRNAs was found in the testes compared to other body organs. The magnitude of circRNAs in testicular samples was significantly greater than that of circRNAs found in other organs, for example, chicken theca cells contained 14,502 circRNAs (Shen et al., 2020), pig pituitaries had 5,148 circRNAs (Ulitsky and Bartel, 2013; Chen et al., 2020), and buffalo fat adipose tissues had 5,141 circRNAs (Huang et al., 2019). We identified 681 coding circRNAs, of which 579 circRNAs were upregulated and 103 were downregulated in immature and mature bull testes. The study composed of Qinchuan cattle identified a total of 21,753 candidate circRNAs, where 2023 circRNAs were downregulated and 2,225 circRNAs were upregulated (Gao et al., 2018). A total of 2,326 differentially expressed circRNAs (DECs) were found in testicular development, of which 1,003 circRNAs were upregulated in adult boar testes and 1,323 circRNAs were downregulated; an analysis of transcriptomic changes in boar testicular tissues revealed an agreement with our results (Zhang et al., 2021). A study of transcriptomic changes in cattle-yak testes showed a tendency toward upregulation rather than downregulation and identified 679 upregulated and 2,281 downregulated genes (Cai et al., 2017). An RNA-seq analysis showed that a total of 10 ,095 genes were substantially differentially expressed in porcine testes, of which 5,199 were upregulated and 4,896 were downregulated in immature and mature porcine testes (Song et al., 2015). Approximately 242 genes were associated with porcine spermatogenesis. The key reason for testis transcriptome profiling is to discover the transcriptional factors that play crucial roles in testis development and spermatogenesis and thus clarify the genetic structure of the testis.

We performed GO annotation and KEGG pathway enrichment analysis to better describe the circRNAs involved in the biological processes of testis development and spermatogenesis. These processes may indicate the roles of circRNAs in the testis or reflect the cellular differences between immature and mature bull testes. In GO annotation, 60 host genes were assigned to spermatogenesis processes, 12 genes to testis development, and 23 host genes were found solely relevant to reproduction phenomena, such as behavior and primary sexual characteristics. Gao et al. (2018) executed the GO annotation, and 44 host genes were assigned to reproduction and the reproductive process, and 25 host genes were associated with spermatogenesis, including *PIWIL1* and the spermatogenesis-associated protein 6 (*SPATA6*). We found different candidate genes by analyzing these GO terms, and some of these genes, such as *ATM* serine/threonine kinase (*ATM*), glycogen synthase kinase 3 beta (*GSK3B*), and cyclin A1 (*CCNA1*), were found to be significantly enriched in the cell cycle pathway and associated with spermatozoa differentiation. The *ATM* protein is associated with low-fertile buffalo bull spermatozoa (M. K et al., 2019); in mouse spermatocytes, the meiotic recombination is initiated by SPO11-induced double-strand breaks *(DSBs)*, and ATM controls this process (Huang et al., 2019; Paiano et al., 2020). *GSK3B* showed significant expression changes across these pubertal stages in the hypothalamus of piglets and is also involved in crucial biological processes that regulate economically relevant traits associated with beef cattle fertility (Fonseca et al., 2018). The GO and KEGG pathway enrichment analyses showed that the DE circRNA genes were associated with pathways, such as the cell cycle, lysine degradation, and tight junction, whereas the top 20 most significant enriched pathways were evaluated in this study. A total of 47 significant signaling pathways in the Qinchuan cattle testis were found, and key host genes including *PIWIL1, DPY19L2, SLC26A8, IFT81, SMC1B, IQCG, TTLL5,* and *ACVR2A* belong to the tight junction, the adherens junction, the TGFβ signaling pathway, progesterone-mediated oocyte maturation, and oocyte meiosis (Gao et al., 2018).

The probable reasons of variation between the findings of the present study and the results of Gao et al. regarding candidate genes and signaling pathways are due to the breed variations. In the current study, we collected samples from Wandong, while Gao et al. used Qinchuan breed testicular tissues for RNA-seq analysis. A study found that many candidate genes were differentially expressed (DE) and also differential alternative spliced in three different cattle breeds, including the Holstein, Jersey, and Cholistani. It suggests that breed-specific gene expression occurs at the transcriptional level and posttranscriptional modifications such as splicing, 5’ capping, and poly A-tail addition. The differentially expressed genes are enriched in KEGG pathways including translation, electron transport, and immune responses (Huang et al., 2012). In the *Bovine* breeds, in Merino vs Poll Dorset, *SLC35A5* and *ITM2C* were identified as key DEGs; both have been reported for their association with conception and embryonic development (Hodge et al., 2021). We found different candidate genes and signaling pathways from the findings of Gao et al. due to variation in age, where they analyzed the testicular tissue in days 7 and in 4 years of age. However, we analyzed the same tissue in the age of 3 months and 3 years. As gene expression is greatly influenced by age, especially genes related to developmental phases, a comparative study was conducted on candidate genes having functional roles in regulation of muscle development at various growth stages in goat. In this study, i) 2 vs 9 months groups were compared and intramuscular fat-linked genes, e.g., *MYH13*, *IFIT1*, *METTL21C*, *SERPINE1*, *EGR1*, and *ESRRG*, were found; ii) 2 vs 24 months groups were matched and significant DEGs *RPS25*, *MYH13*, *COMP*, *LOC102186300*, and *NR4A2* were found; and iii) 9 vs 24 months goat muscles were transcriptionally matched and *PARM1*, *LOC102177715*, *MRF1-like*, *ARID5B*, and *PFKFB3* were found (Lin et al., 2017). Therefore, the candidate genes and signaling pathways are different in these two different studies of similar nature.

In mammalian testes, the cell cycle, lysine degradation, and tight junction and adherens junction pathways and host genes function together to control testis growth, spermatogenesis, and primary sexual activity (Young et al., 2015). The gene expression profiling of testes showed that some lysin degradation pathway genes, including lysine methyltransferase 2E (*KMT2E*) and nuclear receptor-binding SET domain protein 2 (*NSD2*), are active during spermatogenesis. Zhang et al. (2015) reported that the *KMT2E* gene plays key roles in various biological processes, including cell cycle progression, adult hematopoiesis, and spermatogenesis. A vital question raised in developmental biology is that how pluripotent stem cells can give rise to the cells derived from the three germ layers. The *NSD2* gene has a dual role in pluripotency exit and germ layer specification of embryonic stem cells (Tian et al., 2019).

Exonic circRNAs have been shown to act preferentially in post-transcriptional regulation in mice and humans (Bose and Ain, 2018). Similarly, we found that the majority of DE circRNAs identified in this study have putative target miRNA-binding sites, suggesting that most circRNAs are likely to function as miRNA sponges. circRNA–miRNA network analysis further revealed that circRNAs can interact with miRNAs in multiple directions at different rates in the testes, which is consistent with observations in previous studies (Liang et al., 2017). Therefore, when translated, some circRNAs can inhibit or relieve miRNA repression. In this transcriptomic network, we selected 11 DE circRNAs that were significantly matched with 160 miRNAs, wherein the bta-miR-34, bta-miR-532, and bta-miR-204 families were involved in cattle spermatogenesis (Tscherner et al., 2014; Zhang et al., 2015). The related circRNAs may function as miRNA sponges to control the growth of testes and spermatogenesis. The circRNAs act as miRNA sponges, and research on Qinchuan cattle showed that 758 miRNAs matched with significantly differentially expressed circRNAs which play an important role in regulating gene expression in bull testes (Gao et al., 2018). The RT-qPCR validation results indicated that the dynamic expression of the circRNA had a strong interaction with the RNA-seq data. Previous studies have shown that circRNAs exhibit significant developmental stage-precision activity in human testes, suggesting their potential contribution to spermatogenesis (Dong et al., 2016). In rodents, circRNA patterns have been analyzed in three distinct stages, mitotic, meiotic, and postmeiotic germ cells (Lin et al., 2016), and correlated with testis development (Zhou et al., 2018).

## Conclusion

In conclusion, our study presented an extensive analysis of circRNA expression profiles in immature and mature Wandong cattle bull testes. The landscapes of circRNA expression in testes were detected and used for interpreting their regulatory structure in testis development and spermatogenesis in cattle bulls. To date, there is a lack of the genetical data to establish baseline factors related to reproductive performance of local cattle bulls in China. Nevertheless, the insights from this study could lead to a breakthrough in the reproductive efficiency of local cattle resources.

## Data Availability

The datasets presented in this study can be found in online repositories. The names of the repository/repositories and accession number(s) can be found below: https://www.ncbi.nlm.nih.gov/geo/query/acc.cgi?acc=GSE183347, https://www.ncbi.nlm.nih.gov/geo/info/linking.html.

## Abbreviations

GO, gene ontology; KEGG, Kyoto Encyclopedia of Genes and Genomes; DE, differentially expressed; RT-qPCR, real-time quantitative polymerase chain reaction; FPKM, fragments per kilobase of transcript sequence per millions base pairs sequenced; CNCI, coding–non-coding index; CPC, coding potential calculator; nc-RNAs, noncoding RNAs; cds, coding sequence; BP, biological process; CC, cellular components; MF, molecular function; dNTP, deoxyribonucleotide triphosphate; dATP, deoxyadenosine triphosphate; dGTP, deoxyguanosine triphosphate; dUTP, deoxyuridine triphosphate; dCTP, deoxycytidine triphosphate.
