# Supplementary material for: Circular RNA Expression and Regulation Profiling in Testicular Tissues of Immature and Mature Wandong Cattle (Bos taurus)
Source: Front Genet. 2021 Nov 22;12:685541. doi: 10.3389/fgene.2021.685541 (PMC8647812; doi:10.3389/fgene.2021.685541)
Supplement: Supplementary file 1 [file DataSheet1.ZIP › Supplimentry file 1.docx]

| Genes | DE circ-RNAs | Forward 5′→3′ | Reverse 5′→3′ |
| --- | --- | --- | --- |
| *CCNA1* | novel_circ_0003626 | CCCGAAGTAGATGAGTTTGTC | TTCAGCAGCAGGTGTTCC |
| *GSK3B* | novel_circ_0012940 | TCACTGTAACATAGTCCGATTG | GTCCAGCACCAGATTAAGATAG |
| *NSD2* | novel_circ_0027610 | GCCACCACCCTCACCTTTAG | CCCTGCGTCCTCTTCGTG |
| *KMT2E* | novel_circ_0024951 | GGAAGATGGGTTTGTGGATG | GTTCGCCTCTGGTAAATTCG |
| *SUCLG2* | novel_circ_0015698 | TTATTGAAGGGATAAAGGACAGC | AACCGCTACTGAAGACACC |
| *ATM* | novel_circ_0006810 | TTGTGCTGCTCATTTTACCG | ATCCACAGGCTTGTATCATCC |
| *QKI* | novel_circ_0032892 | TTTAATTTTGTTGGGAGAATCCTTGGAC | TCCGTACTCTGCTAATTCTGGGCAAACT |
| *HOMER1* | novel_circ_0000114 | AATACAAGAACTGGAGGAGACGCTGAAG | AGCTGCTGCTTCCATTGTTTCACATTGG |
| *SNAP91* | novel_circ_0031845 | TTTGCTTGTTACAACGATGGAGTGATTA | GATGTGTAGTGACTAAAGCTTTGAATAC |

**Table S2. Primers used for RT-qPCR validation of circ-RNA-seq data**

*DE circ-RNAs represent differentially expressed circular RNAs
