# Supplementary material for: Circular RNA Expression and Regulation Profiling in Testicular Tissues of Immature and Mature Wandong Cattle (Bos taurus)
Source: Front Genet. 2021 Nov 22;12:685541. doi: 10.3389/fgene.2021.685541 (PMC8647812; doi:10.3389/fgene.2021.685541)
Supplement: Supplementary file 1 [file DataSheet1.ZIP › Supplimentry file 2.docx]

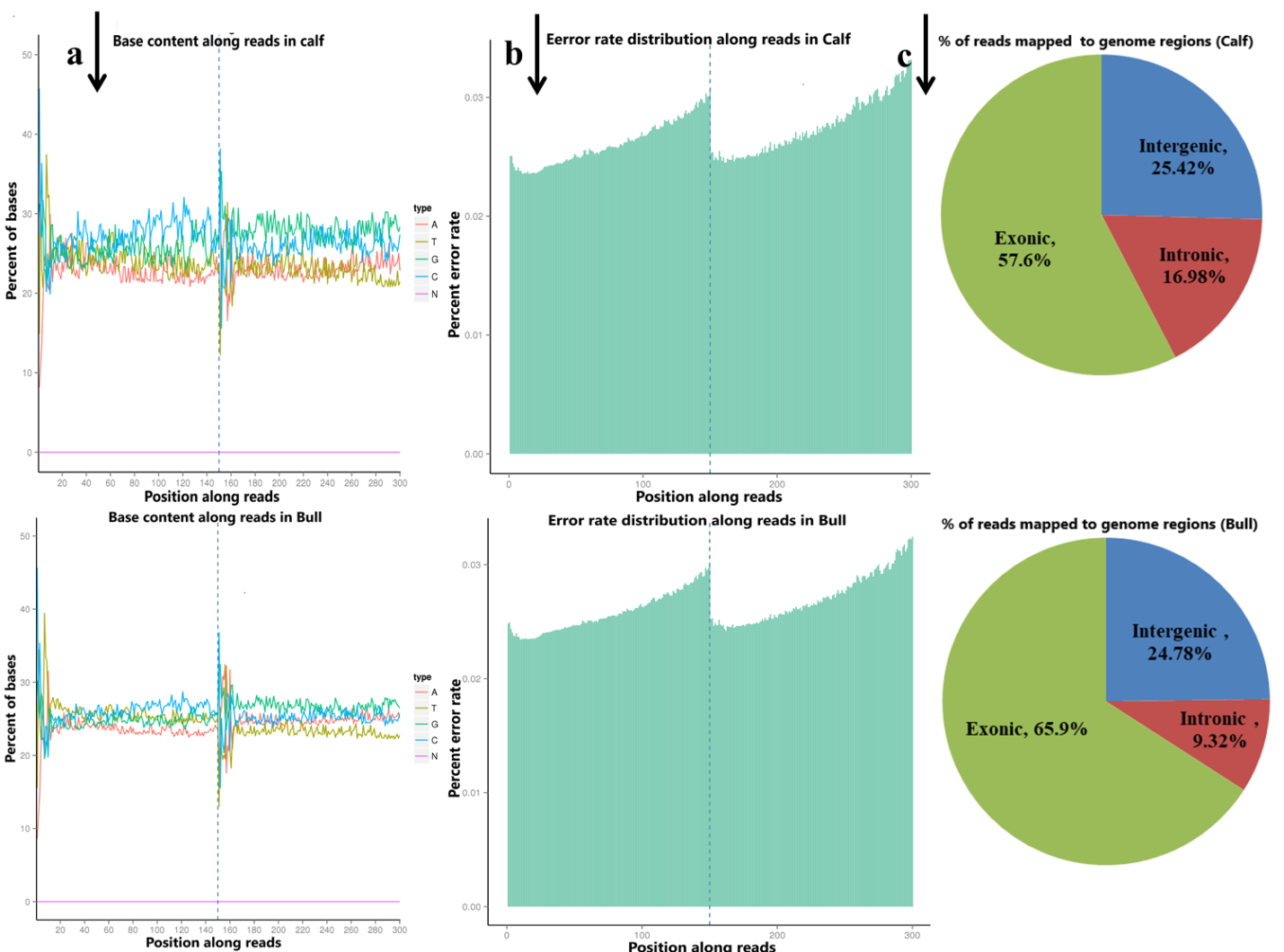


**Additional file 1.** The quality summary of clean reads used in subsequent analysis were obtained after original data filtering, sequencing error rate checking and GC content distribution checking. (a) The abscissa shows the base position of reads, and the ordinate is the proportion of single base, whereas different colors represent different base types. (b) The abscissa represents the bases position of reads, and the ordinate is the average error rate of all reads at this location. 0~150 bp to the left of the dotted line is the error rate distribution of read1, while 150~300 bp to the right is the error rate distribution of read2. (c) The percentage of the total number of clean reads mapped to the reference genome. The proportions of reads in the exon region, intron region and intergene region of the genome were calculated.
