# Supplementary material for: Circular RNA Expression and Regulation Profiling in Testicular Tissues of Immature and Mature Wandong Cattle (Bos taurus)
Source: Front Genet. 2021 Nov 22;12:685541. doi: 10.3389/fgene.2021.685541 (PMC8647812; doi:10.3389/fgene.2021.685541)
Supplement: Supplementary file 1 [file DataSheet1.ZIP › Supplimentry file 3.docx]

Additional file 2. Raw data quality summery.

| **Sample_name** | **Raw_reads** | **Clean_reads** | **Raw_bases (G)** | **Clean_bases (G)** | **Error rate(%)** | **Q20(%)** | **Q30(%)** | **GC_content(%)** |
| --- | --- | --- | --- | --- | --- | --- | --- | --- |
| Bull_1 | 142165686 | 140666436 | 21.32 | 21.10 | 0.03 | 97.40 | 92.90 | 52.53 |
| Bull_2 | 141654378 | 139622682 | 21.25 | 20.94 | 0.03 | 97.42 | 92.94 | 51.48 |
| Bull_3 | 117468592 | 115051574 | 17.62 | 17.26 | 0.03 | 97.38 | 92.87 | 51.98 |
| Calf_1 | 142605328 | 140436238 | 21.39 | 21.07 | 0.03 | 97.50 | 93.11 | 53.57 |
| Calf_2 | 115463518 | 112484344 | 17.32 | 16.87 | 0.03 | 97.23 | 92.64 | 53.34 |
| Calf_3 | 118785371 | 116004150 | 17.82 | 17.40 | 0.03 | 97.25 | 92.53 | 47.09 |
